# 4. INNOVATIVE APPROACHES TO EARLY IDENTIFICATION AND TREATMENT: USING MOBILE HEALTH TECHNOLOGY TO IMPROVE OUTCOMES IN PSYCHOSIS

**DOI:** 10.1093/schbul/sby014.007

**Published:** 2018-04-01

**Authors:** Laura Tully

**Affiliations:** University of California, Davis

## Abstract

**Overall Abstract:** Smartphone and internet based applications that promote symptom tracking, treatment engagement, and self-management have the potential to improve mental health outcomes and reduce cost of care. This is especially important in the treatment of psychosis, as long-term clinical outcomes to commonly available treatments remain poor and financial costs are high. The speakers in this symposium will present novel approaches using mobile health technology to promote rapid identification and referral (Dr. Niendam), access to treatment (Dr. Hidalgo-Mazzei), treatment engagement and symptom tracking (Dr. Tully), and functional recovery (Dr. Alvarez-Jimenez) for individuals experiencing psychosis.

Dr. Niendam will present initial results of a community-based cluster-randomized controlled trial aiming to increase identification rates of individuals with psychosis and reduce Duration of Untreated Psychosis. Twenty-two school, community, and primary care sites in Sacramento, California were randomized to either standard community education and clinician-based referral versus standard education plus electronic (tablet) screening for psychosis symptoms. Results show electronic screening is feasible across various community settings and significantly increases identification rates compared to clinician-based identification alone.

Dr. Hidalgo-Mazzei will present data examining the feasibility of delivering a psychoeducational treatment program that promotes self-management in bipolar disorder. The SIMPLe platform is accessible from any internet enabled device and provides symptom monitoring and personalized psychoeducation content in Spanish, Italian, and French. Data from a large open trial with over 300 participants from across the globe demonstrate how mobile technologies can increase access to care by extending effective interventions to many people at low cost.

Dr. Tully will present data on the feasibility, validity, and predictive utility of a consumer smartphone application (“app”) plus provider web-based Dashboard as an add-on treatment tool in Early Psychosis outpatient programs in Northern California. Data demonstrate that consumers and providers in community-based outpatient clinics are responsive to integrating smartphone technology into treatment services. Consumers willingly use the app to track their symptoms; symptom data gathered via the app appears to be a valid reflection of symptoms experienced over time and can predict symptom exacerbations two weeks later.

Dr. Alvarez-Jimenez will present data demonstrating the feasibility, acceptability, and efficacy of two novel online social media based platforms designed to promote functional recovery in Ultra High Risk and First Episode Individuals. Results indicate online social media platforms are safe, engaging, and improve social functioning in both populations – a domain that is often neglected in most treatment approaches.

Chantel Garrett is the founder of Strong365.org, a website providing consumer and family-focused psychoeducation materials related to psychosis in 103 languages. She also has lived experience as a family member of a loved one with schizophrenia. As discussant, she will speak from her expertise as both a developer and consumer of internet-based and mobile technologies to elucidate how mobile health materials can impact provision of mental health care, and facilitate discussion of the barriers and future directions for the field. Implementation of technology-based care across diverse cultures and languages will also be discussed.

